# The Effect of Perceived Environmental Uncertainty on University Students’ Anxiety, Academic Engagement, and Prosocial Behavior

**DOI:** 10.3390/bs13110906

**Published:** 2023-11-03

**Authors:** Ting Kong, Shuang Zeng

**Affiliations:** 1 Business School, University of Shanghai for Science and Technology, Shanghai 200093, China; kongting@usst.edu.cn; 2 College of Foreign Languages, University of Shanghai for Science and Technology, Shanghai 200093, China

**Keywords:** perceived environmental uncertainty, anxiety, academic engagement, prosocial behavior, intolerance of uncertainty, university students

## Abstract

Uncertainty, as the predominant characteristic of the contemporary landscape, poses significant challenges and exerts profound influence on individual decision making and behaviors; however, there remains a limited understanding of its impact on university student behavior. Building upon the uncertainty management theory, this study presents a conceptual framework to investigate the impact of perceived environmental uncertainty on university students’ anxiety levels and behaviors, including academic engagement and prosocial behavior. Additionally, our model proposes that the intolerance of uncertainty moderates a mediating effect on anxiety. These hypotheses are empirically tested using a sample of 221 Chinese university students. The results reveal a positive relationship between perceived environmental uncertainty and anxiety among university students; subsequently, anxiety exerts a negative influence on both academic engagement and prosocial behavior. Furthermore, we find that anxiety serves as a psychological mediator between perceived environmental uncertainty and both academic engagement and prosocial behavior. This research also underscores the significance of the intolerance of uncertainty in shaping university students’ involvement in academic pursuits when confronted with anxiety stemming from perceived environmental uncertainty. Consequently, these findings have practical implications for facilitating university students’ adaptive coping strategies in uncertain contexts and mitigating the negative effects of anxiety on their behavioral responses.

## 1. Introduction

University students who are in the phase of emerging adulthood undergo critical transitions characterized by transformative changes, cognitive dissonance, exploratory behavior, and psychological maturation [1,2]. Consequently, they represent a population subgroup whose behaviors and decision-making processes exhibit heightened susceptibility to the influence of their living environment, particularly in situations characterized by environmental uncertainty. In contemporary society, university students are confronted with a multitude of environmental uncertainties encompassing economic, political, and social dimensions. This is particularly accentuated during crises, such as the climate change emergency, economic downturn, and the COVID-19 pandemic. Persistent climate change is acknowledged to present a variety of uncertainties and threats to human health, including mental and emotional health [3]. Climate change can trigger adverse emotional responses, such as exacerbating climate anxiety, which is particularly prevalent among young adults [4,5]. The current global economy is undergoing a substantial deceleration. The extensive implications of an economic downturn are far-reaching, including a pronounced impact on mental health [6]. Given the distinct position of university students in the employment landscape, they confront the dual challenges of graduation and job acquisition. The stagnation of labor mobility, the reduction in new job opportunities, and the escalated uncertainty of employment have all contributed to the anxiety of university students [7]. Furthermore, the COVID-19 pandemic has introduced an unprecedented level of uncertainty and unpredictability into the global economy and society, profoundly affecting various aspects of people’s lives [8]. The implementation of a range of control measures, such as lockdowns, social distancing policies, school closures, and online learning, has forced large numbers of university students to home study remotely in a much more uncertain environment. This shift in educational modality has resulted in increased feelings of isolation, loneliness, and anxiety among students, which have significantly impacted their behavioral patterns [9]. However, the impact of increased environmental uncertainty on university students’ mental health and subsequent behavior remains insufficiently documented and necessitates further investigation.

The profound Impact of perceived environmental uncertainty on the behavior of university students raises a number of important questions that remain unanswered. First, does perceived environmental uncertainty have a significant impact on the mental health of university students, particularly in terms of anxiety? Anxiety, a psychological phenomenon characterized by feelings of fear and apprehension in unfamiliar and unpredictable situations [10], is widely acknowledged as a pivotal determinant of mental health among today’s university students. Moreover, it serves as a specific psychological mechanism that influences decision-making behavior in this population. According to the theory of uncertainty management, anxiety often arises as a consequence of uncertainty [11], which is detrimental to individual well-being [12]. Furthermore, several anecdotal reports from healthcare professionals suggest that the COVID-19 pandemic engenders a substantial increase in individual anxiety due to the prevailing uncertainty [13]. A number of national surveys provide evidence of negative emotions, such as climate anxiety, induced by climate change and its attendant uncertainties [3,5]. However, the impact of perceived environmental uncertainty on university students’ anxiety remains unclear. Further research is needed.

Second, how does perceived environmental uncertainty affect the behavior of university students, particularly their academic engagement and prosocial behavior? The presence of uncertainty in the environment can induce emotional distress in individuals, leading to significant changes in their decision making and behaviors [14]. Scholars have posited that an uncertain environment can engender a potential threat by amplifying the anxiety levels of university students [9,15], thereby exerting a detrimental impact on their behavioral patterns. However, some scholars argue that uncertainty fosters an individual’s tendency to adopt a broader perspective on life, overcome obstacles, and exhibit greater prosocial tendencies. The current understanding of the influence of perceived environmental uncertainty on various behaviors (such as academic engagement and prosocial behaviors) among university students remains inadequate. Consequently, there is an urgent need for further exploration of these effects. Therefore, this study aims to elucidate the underlying psychological mechanisms through which anxiety mediates the impact of perceived environmental uncertainty on both academic engagement and prosocial behavior.

Third, under what specific circumstances does anxiety induced by perceived environmental uncertainty exert a more pronounced detrimental influence on university students’ academic engagement and prosocial behavior? Research on uncertainty management has shown that better coping strategies can mitigate the negative effects of anxiety associated with uncertainty [16]. The intolerance of uncertainty encompasses the cognitive, emotional, and behavioral responses to uncertain events in life; it reflects an individual’s incapacity to effectively navigate uncertain situations or unpredictable events [17]. Individuals who have a high level of intolerance of uncertainty are more likely to perceive uncertainty as threatening and distressing, thereby increasing the negative impact of the resulting anxiety. Therefore, in order to investigate the coping strategies employed by university students in response to environmental uncertainty, it is crucial to understand the impact of their intolerance of uncertainty on the association between anxiety induced by perceived environmental uncertainty and university students’ academic engagement and prosocial behavior.

Given the aforementioned research gaps in the field, this present study aims to construct and validate a model that delineates the mechanisms through which perceived environmental uncertainty impacts university students’ anxiety, academic engagement, and prosocial behavior. Furthermore, we examine the role of intolerance of uncertainty as a coping mechanism to mitigate the potential negative effects of environmental uncertainty. Thus, this study contributes to existing theory and research in three ways. First, our study focuses on examining the core principle of uncertainty management theory through the lens of perceived environmental uncertainty [11], thus extending this theory by applying it to the university context, an area that has received relatively little attention in previous studies. Second, we advance scholarly understanding by proposing a comprehensive conceptual framework that elucidates the underlying psychological mechanisms through which perceived environmental uncertainty affects the psychological well-being and critical behaviors of university students. In particular, our study identifies anxiety as a critical mediator that accounts for the negative effects of perceived environmental uncertainty while also highlighting how such uncertainty can simultaneously influence both academic engagement and prosocial behavior. Third, our study examines the moderating role of intolerance of uncertainty in influencing the negative effects of anxiety associated with environmental uncertainty on academic engagement and prosocial behavior. Thus, we contribute to the field of uncertainty management research by emphasizing the necessity of enhancing students’ tolerance for uncertainty, thereby unearthing coping mechanisms that mitigate the negative effects of environmental uncertainty on the mental health and associated behavioral patterns of this demographic.

## 2. Literature Review and Research Hypotheses

**Figure 1:**
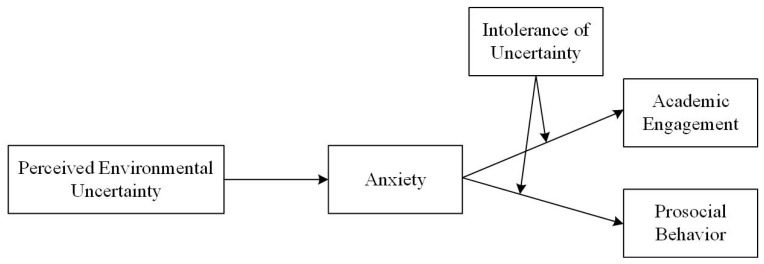
The theoretical conceptual model.

In the following section, we propose a theoretical framework that elucidates the psychological mechanisms through which anxiety arising from perceived environmental uncertainty influences university students’ academic engagement and prosocial behavior while also considering the moderating influence of intolerance of uncertainty. The comprehensive conceptual model is illustrated in Figure 1.

### 2.1. Perceived Environmental Uncertainty and Anxiety

Environmental uncertainty refers to the external environment and results from an individual’s inability to accurately predict the future trajectory of environmental factors [18], including political, economic, governmental, cultural, and discontinuous uncertainties [19]. However, due to its subjective nature, uncertainty encompasses multiple perspectives as it involves individual interpretations of events and resources [20]. Perceived environmental uncertainty, as defined by Milliken [18], refers to the degree to which individuals perceive the direction of change in the environment, the potential impact of that change on their activities, and the best way to respond.

Anxiety arises when there is uncertainty about future events and a lack of clarity about effective preventive or mitigating actions that individuals can take [21]. In this paper, we specifically focus on examining general state anxiety, which refers to distress and/or physiological arousal experienced in response to stimuli such as novel situations and potentially undesirable outcomes [10]. State anxiety is likely to manifest in uncertain environments, such as those contexts associated with the climate change crisis, economic downturn, and the COVID-19 pandemic.

The study of anxiety and its effects on performance is often a priority in research on uncertainty [22]. Environments that are perceived as highly uncertain are likely to be viewed as inherently risky, where even a few mistakes could lead to significant consequences. Obviously, such an environment would tend to elicit a heightened sense of anxiety. Uncertainty management theory provides an appropriate theoretical framework to understand the potential impacts of perceived environmental uncertainty on anxiety. According to this theory, uncertainty often provokes anxiety [11]. In the face of unrelenting uncertainty, individuals experience increasing discomfort, distress, and anxiety about their current circumstances and future prospects, culminating in an escalation of anxiety. For instance, the emotional impacts of climate change are primarily borne by young individuals despite their minimal contribution to the crisis. These individuals possess limited opportunities yet invaluable perspectives for influencing climate action. Consequently, their anxiety levels are heightened due to concerns regarding climate change and the uncertainty of future environmental conditions [5]. During an ongoing economic downturn, the uncertainty surrounding the employment landscape continues to escalate, thereby exacerbating the level of anxiety experienced by university students seeking employment [7]. Furthermore, the amplified levels of uncertainty and perceived danger during the COVID-19 pandemic were exacerbated by continuous confirmed cases, inadequate information, and the escalating disruption of personal lives. Consequently, anxiety emerged as individuals faced persistent threats that could not be alleviated by avoidance or evasion [23]. Thus, we propose the following:

**Hypothesis** **1.** *Perceived environmental uncertainty is positively related to university students’ anxiety*.

### 2.2. Anxiety, Academic Engagement, and Prosocial Behavior

Anxiety, in and of itself, is an extremely unpleasant aversive state. However, its insidious nature lies in the profound impact it has on individual cognition and attention, thereby influencing decision making and behaviors [23]. When faced with uncertainty, individuals carefully evaluate their available resources to initiate adaptive coping responses [24]. Existing theoretical frameworks and empirical studies suggest that the experience of anxiety triggers defense mechanisms in the form of a fight-or-flight response aimed at avoiding potential threats. The fight response is activated when the threat is perceived as surmountable, while the flight response is triggered when the threat is perceived as difficult to overcome [13,25]. The perceived environmental uncertainty triggers anxiety among university students, which is likely to elicit flight responses. Consequently, this may lead to a decline in academic engagement and a reduction in prosocial behaviors across both academic and community domains.

According to uncertainty management theory, individuals exhibit different responses and behaviors to perceived environmental uncertainty and associated anxiety [11,16]. In general, anxiety stemming from perceived environmental uncertainty can elicit self-protective, avoidant, and defensive behaviors [26] as a means for individuals to function effectively without being overwhelmed by anxiety [14]. One strategy for self-protection in the workplace is to reduce personal engagement—that is, to disengage from work roles both physically and cognitively while also reducing emotional involvement [27]. Similarly, university students use this approach to reduce their academic engagement. Academic engagement refers to behaviors that are vigorous, dedicated, and deeply involved in study-related activities, reflecting a positive and fulfilling academic disposition [28]. When students experience heightened anxiety due to perceived environmental uncertainty, they tend to experience confusion about their future career plans and developmental prospects, unease about their current learning goals, and potential setbacks in the form of slowed learning progress and increased distraction [29]. Consequently, this leads to a decline in learning status and performance, which ultimately results in reduced academic engagement. Accordingly, we argue that anxiety may impede the learning behavior of university students. Thus, we propose the following:

**Hypothesis** **2a.** *Anxiety is negatively related to academic engagement*.

According to the theory of uncertainty management, uncertainty-induced anxiety serves as a motivating force that prompts individuals to effectively cope with uncertainty [11]. Furthermore, an individual’s sense of personal value acts as a crucial coping resource. Anxiety, an unpleasant emotional state, prompts individuals to avoid or escape from anxiety-provoking situations [10,30]. Individuals with high levels of anxiety may exhibit a tendency to decrease their involvement in community volunteer activities. Consequently, anxiety evokes flight responses among university students, leading to a decline in prosocial behavior, which acts as a coping mechanism for managing anxiety. This study defines prosocial behavior as an individual’s volunteering, i.e., offering one’s time or skills to a volunteer group or organization in a planned activity, with the purpose of better helping others [31]. An increase in anxiety due to perceived environmental uncertainty may lead to a decrease in prosocial behavior among university students because they fear that engaging in community service may increase the risk of contagion of such anxiety. Conversely, a decrease in anxiety induced by perceived environmental uncertainty may lead to an increase in prosocial behavior among university students as they become more motivated and capable of helping others within the community. Empirical evidence also suggests that the presence of anxiety alone does not significantly contribute to the manifestation of prosocial behavior unless individuals perceive a shared sense of humanity with those in need [32]. Thus, we propose the following:

**Hypothesis** **2b.** *Anxiety is negatively related to prosocial behavior*.

### 2.3. Mediating Effect of Anxiety

Precipitated by environmental uncertainty, anxiety exerts a pivotal influence on the formation of behavioral patterns in university students. Uncertainty management theory posits that an uncertain situation tends to induce an anxious state [11], which has detrimental effects on individuals’ behavior. Empirical research has also shown that anxiety serves as a determinant of the detrimental consequences of perceived uncertainty on individuals’ well-being and performance [14,23]. Consequently, we propose that anxiety serves as a mediating factor in the association between perceived environmental uncertainty and university students’ behavior, including both academic engagement and prosocial behavior.

In the current VUCA era, university students continue to face a highly uncertain environment characterized by volatility, uncertainty, complexity, and ambiguity. Individuals who perceive higher levels of environmental uncertainty tend to experience higher levels of anxiety [16]. Conversely, individuals who perceive lower levels of environmental uncertainty are less likely to be disturbed or influenced, resulting in lower levels of anxiety. In addition, anxiety can negatively affect students’ academic engagement and prosocial behavior. The more anxious students are, the less likely they are to engage in academic and prosocial activities. Therefore, university students’ perceptions of environmental uncertainty result in an increase in anxiety, which subsequently leads to a decrease in academic engagement and prosocial behavior. Thus, we propose the following:

**Hypothesis** **3a.** *Anxiety mediates the relationships between perceived environmental uncertainty and academic engagement. In particular, the perceived environmental uncertainty in university students leads to an increase in anxiety, which subsequently leads to a decrease in academic engagement*.

**Hypothesis** **3b.** *Anxiety mediates the relationships between perceived environmental uncertainty and prosocial behavior. In particular, the perceived environmental uncertainty in university students leads to an increase in anxiety, which subsequently leads to a reduction in prosocial behavior*.

### 2.4. Moderating Effect of Intolerance of Uncertainty

According to uncertainty management theory, the negative consequences of anxiety arising from uncertainty can be mitigated if individuals possess robust psychological resources and capabilities [13], such as a low level of intolerance of uncertainty. According to Dugas and Gagnon [33], the intolerance of uncertainty refers to the tendency to perceive uncertain situations or outcomes as intolerable and threatening, regardless of the actual likelihood of events occurring. The intolerance of uncertainty represents an individual’s ability to cope with uncertainty, indicating their inability to effectively navigate ambiguous situations or unpredictable events [17]. Therefore, individuals can use coping strategies that focus on their ability to mitigate the negative effects of environmental uncertainty on anxiety and behavior. University students who exhibit lower levels of intolerance of uncertainty are better equipped to effectively reduce the negative effects of anxiety on their behavior.

First, we propose that the intolerance of uncertainty moderates the association between perceived environmental uncertainty, anxiety, and academic engagement. Compared to university students with higher levels of intolerance of uncertainty, those with lower levels demonstrate greater effectiveness in mitigating the negative impact of anxiety induced by perceived environmental uncertainty on academic engagement. On the one hand, university students with a lower intolerance for uncertainty are more likely to acknowledge their uncertainties and concerns [17], which allows them to endure or minimize the impact of uncertainty, show greater confidence in managing the resulting anxiety, and demonstrate a greater willingness to invest appropriately in their studies. On the other hand, university students with lower intolerance of uncertainty tend to prioritize their own needs and actively seek additional resources, thereby gaining greater autonomy in managing challenging situations. This enhanced sense of control subsequently mitigates the negative effects of anxiety on university students’ academic engagement [27]. Integrating the above statements that highlight how perceived environmental uncertainty intensifies anxiety while a lower intolerance of uncertainty diminishes its negative influence on academic engagement, we propose the following:

**Hypothesis** **4a.** *The negative indirect relationship between perceived environmental uncertainty and academic engagement via anxiety is strengthened when the intolerance of uncertainty is higher compared to when the intolerance of uncertainty is lower*.

Second, we propose that the intolerance of uncertainty moderates the association between perceived environmental uncertainty, anxiety, and prosocial behavior. Compared to individuals with a high intolerance of uncertainty, university students with a low intolerance of uncertainty demonstrate greater effectiveness in mitigating the negative impact of anxiety arising from perceived environmental uncertainty on prosocial behavior. In particular, in contrast to those with a high intolerance of uncertainty, university students with a low intolerance of uncertainty exhibit enhanced adaptability to environmental uncertainty, resulting in reduced anxiety and an increased sense of humanity and community. As a result, they are more likely to engage in problem-solving efforts that specifically address uncertainty by volunteering to help a greater number of people [14]. Therefore, the perceived environmental uncertainty induces anxiety, while lower levels of intolerance of uncertainty mitigate the detrimental effects of anxiety on prosocial behavior. Thus, we propose the following:

**Hypothesis** **4b.** *The negative indirect relationship between perceived environmental uncertainty and prosocial behavior through anxiety is strengthened when intolerance of uncertainty is higher compared to when intolerance of uncertainty is lower*.

## 3. Methods

### 3.1. Sample and Procedure

An online survey, widely used in behavioral and psychological research, was used to collect data from several universities in eastern China [1]. The survey of the empirical study was conducted in February 2023. A cover letter was included to inform participants of the scientific nature of the survey and to ensure the confidentiality of their responses. Participants voluntarily completed the questionnaire, which was designed to assess their perceived environmental uncertainty, anxiety, academic engagement, prosocial behavior, and intolerance of uncertainty. The survey required approximately 10 min to complete. The final sample size was 221 participants. Within this final sample, 55.7% were female, 58.4% were master’s degree candidates, and the mean age was 23 years (SD = 3.14). Participants represented a variety of disciplines, including engineering, science, management, and business.

### 3.2. Measures

All English measures were translated into Chinese by two bilingual researchers using a rigorous translation-back-translation process [34]. The disagreements and inaccuracies in the translation were resolved through further discussion, resulting in the development of a Chinese-language questionnaire. The questionnaire was piloted among 20 Chinese university students and revised and finalized accordingly. Unless otherwise noted, all measures used a 7-point Likert scale ranging from 1 (strongly disagree) to 7 (strongly agree).

#### 3.2.1. Perceived Environmental Uncertainty

The measurements of perceived environmental uncertainty were derived from the work of Waldman, Ramirez [35] and adjusted to fit the specific needs of this study. The responding students were required to characterize the external environment in which their universities operate, including not only the economic landscape but also the social, political, and technological dimensions. Sample items included statements such as “Highly dynamic, characterized by rapid changes in technical, economic, and cultural aspects”, “Significantly risky, where a single misstep can lead to a decline in university ranking”, and “Extremely stressful, exacting, hostile; challenging to maintain stability”. Reliability analysis yielded a Cronbach’s alpha coefficient of 0.734.

#### 3.2.2. Anxiety

The anxiety measures were derived from studies conducted by Watson, Clark [36], and Lian, Li [22]. Participants were asked to indicate their level of experience with three different emotions: anxiety, worry, and tension. All items were rated on a 7-point Likert scale ranging from 1 (not at all) to 7 (very much). Reliability analysis revealed a Cronbach’s alpha coefficient of 0.947.

#### 3.2.3. Academic Engagement

The Chinese version of the Utrecht Work Engagement Student Scale (UWES-S), developed by Schaufeli, Salanova [28], was used to assess the academic engagement of university students in this study. The scale consists of 17 items in three subscales: vigor, dedication, and absorption. Vigor consists of six items, as exemplified by the sample item “When I’m engaged in my student work, I experience a surge of energy”; dedication consists of five items, as exemplified by the sample item “I perceive my learning as meaningful and purposeful”; and absorption consists of six items, as exemplified by the sample item “It’s difficult for me to detach myself from my academic pursuits”. All items were assessed using a 7-point frequency scale ranging from 1 (never) to 7 (always). Higher scores on each subscale indicate higher levels of academic engagement. The coefficients of Cronbach’s alpha were found to be satisfactory: 0.942 for vigor, 0.971 for commitment, and 0.928 for absorption.

#### 3.2.4. Prosocial Behavior

Prosocial behavior was measured using five items adapted from Rodell [31]. Respondents were asked to evaluate their level of involvement in volunteer activities over the past year. Sample items include “I dedicate my time to support a volunteer organization”, and “I apply my skills in ways that benefit a volunteer group”. Reliability analysis revealed a Cronbach’s alpha coefficient of 0.928.

#### 3.2.5. Intolerance of Uncertainty

The Intolerance of Uncertainty Scale—Short Form (IUS-12), which was developed by Carleton, Norton [37], was used to assess participants’ intolerance of uncertainty by measuring their aversion to perceived uncertain information. The scale consists of 12 items and includes two dimensions: prospective anxiety and inhibitory anxiety. Sample items include “Unforeseen events worry me a lot”, “I always want to know about my future prospects”, and “When I am uncertain, I can’t function very well”. Notably, this scale has demonstrated robust reliability and validity in the Chinese context, as evidenced by a Cronbach’s alpha coefficient of 0.898.

#### 3.2.6. Control Variables

To mitigate the impacts of exogenous factors, we have incorporated four control variables into our research. These controls encompass participants’ demographic characteristics, including gender, age, educational level, and major.

## 4. Results

### 4.1. Descriptive Statistics and Correlations

Descriptive statistics and correlations between perceived environmental uncertainty, anxiety, academic engagement, prosocial behavior, and intolerance of uncertainty are presented in Table 1. Perceived environmental uncertainty showed a positive relationship with anxiety (r = 0.17, *p* < 0.05). Anxiety showed negative associations with both academic engagement (r = −0.45, *p* < 0.01) and prosocial behavior (r = −0.20, *p* < 0.01) while showing a positive correlation with the intolerance of uncertainty (r = 0.43, *p* < 0.01). In addition, there was a positive correlation between academic engagement and prosocial behavior (r = 0.41, *p* < 0.01), while a negative correlation between academic engagement and intolerance of uncertainty (r = −0.20, *p* < 0.01). These results provide the statistical basis for testing our hypothesized model.

### 4.2. Path Analysis

Prior to conducting our analyses, we performed a confirmatory factor analysis (CFA) to assess the discriminant validity of the scale measures [38]. Our proposed five-factor model showed satisfactory fit indices: *χ*^2^ = 1237.48, df = 542, comparative fit index (CFI) = 0.91, root mean square error of approximation (RMSEA) = 0.08, and standardized root mean residual (SRMR) = 0.05. Moreover, it outperformed alternative models in which (a) perceived environmental uncertainty and anxiety were combined, (b) academic engagement and prosocial behavior were combined, and (c) all items were aggregated together (see Table 2).

We employed a path analysis in Mplus 8 [39] to test our hypotheses. Following established guidelines [40], we centered anxiety and intolerance of uncertainty prior to calculating their interaction term. The results of the path analyses are presented in Table 3.

Hypothesis 1 posited a positive relationship between perceived environmental uncertainty and university students’ anxiety. As shown in Model 1 of Table 3, we observed a significant positive effect of perceived environmental uncertainty on anxiety (B = 0.37, SE = 0.07, *p* < 0.001), supporting Hypothesis 1.

Hypothesis 2a and 2b proposed that anxiety exhibits a negative correlation with both academic engagement and prosocial behavior. The results of Model 1 provide support for these hypotheses, as we found that anxiety was negatively related to both academic engagement (B = −0.29, SE = 0.04, *p* < 0.001) and prosocial behavior (B = −0.21, SE = 0.07, *p* < 0.01).

Hypotheses 3a and 3b postulated that anxiety mediates the relationship between perceived environmental uncertainty and academic engagement as well as between perceived environmental uncertainty and prosocial behavior. To assess mediation, we conducted a Monte Carlo bootstrap simulation with 5000 replications to generate bias-corrected 95% confidence intervals (CIs) for our indirect effects. Consistent with the recommendations of Preacher and Hayes (2004), we included the direct effects of anxiety on each dependent variable when testing for mediation and allowed for covariance among the residuals of our dependent variables. The bootstrap results indicated that perceived environmental uncertainty had a negative indirect effect on academic engagement (estimate = −0.11, 95% CI [−0.221, −0.056]) and prosocial behavior (estimate = −0.07, 95% CI [−0.150, −0.013]) through its effect on anxiety. Anxiety fully mediated these relationships. Consequently, university students experiencing higher levels of perceived environmental uncertainty exhibited reduced academic engagement and prosocial behavior due to increased anxiety. Hypothesis 3a and 3b were supported.

**Figure 2:**
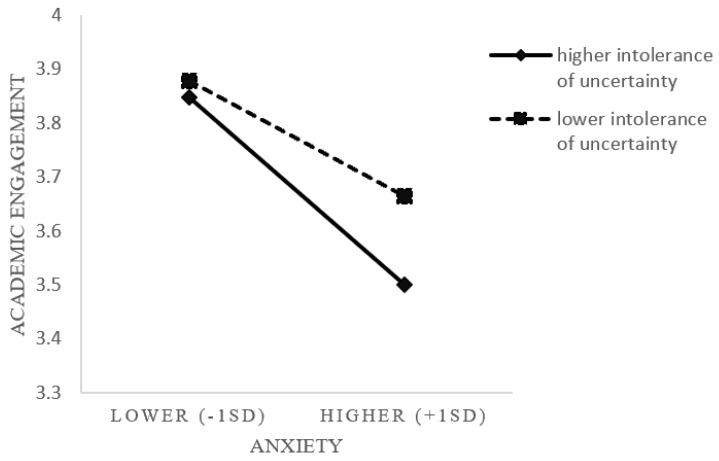
The interaction effect of intolerance of uncertainty and anxiety on academic engagement.

Hypothesis 4 stated that the moderating role of the intolerance of uncertainty would negatively influence the indirect effects of perceived environmental uncertainty on (a) academic engagement and (b) prosocial behavior through anxiety. The results in Model 2 indicated that the interaction of intolerance of uncertainty and anxiety was negatively related to academic engagement (B = −0.10, SE = 0.05, *p* < 0.05). Further examination via simple slope analyses revealed that when intolerance of uncertainty was higher, the association between anxiety and academic engagement became more negative (B = −0.35, *p* < 0.001) compared to when it was lower (B = −0.21, *p* < 0.001), as shown in Figure 2. Additionally, our results indicated that there was a significant and negative indirect relationship between perceived environmental uncertainty and academic engagement via anxiety when intolerance of uncertainty was higher (B = −0.13, 95% CI [−0.221, −0.048]) but not significant when it was lower (B = −0.08, 95% CI [−0.158, 0.003]), supporting Hypothesis 4a. However, no significant effect was observed between the interaction and prosocial behavior (B = −0.10, SE = 0.07, n.s.), indicating that the intolerance of uncertainty does not moderate the indirect relationship between perceived environmental uncertainty and prosocial behavior through anxiety. Therefore, Hypothesis 4b was not supported.

As for control variables, the results in Model 1 revealed a negative association between age and academic engagement (B = −0.38, SE = 0.14, *p* < 0.01), while education level showed a positive association with academic engagement (B = 0.61, SE = 0.19, *p* < 0.01). The association between gender and academic engagement (B = −0.18, SE = 0.13, n.s.), between major and academic engagement (B = 0.17, SE = 0.14, n.s.), between gender and prosocial behavior (B = 0.14, SE = 0.18, n.s.), between age and prosocial behavior (B = −0.23, SE = 0.20, n.s.), between education level and prosocial behavior (B = 0.38, SE = 0.26, n.s.), and between major and prosocial behavior (B = −0.04, SE = 0.20, n.s.) were non-significant.

## 5. Discussion and Conclusions

### 5.1. Theoretical Implications

By leveraging uncertainty management theory, this study develops a model to clarify the effects of perceived environmental uncertainty on anxiety, academic engagement, and prosocial behavior. Accordingly, this study unravels the underlying psychological mechanisms through which perceived environmental uncertainty affects anxiety and essential behaviors of university students. The findings of this present study suggest that perceived environmental uncertainty has a positive influence on the escalation of anxiety in university students. When faced with an ambiguous environment, perceived environmental uncertainty triggers feelings of anxiety in this specific population. These findings are consistent with those of previous studies that individuals experience unpleasant anxiety when they perceive external uncertainty [13,23].

Furthermore, our findings reveal a significant negative relationship between anxiety levels and university students’ academic engagement and prosocial behavior, thereby expanding our understanding of the antecedents of response behavior in both academic and community settings. Building on previous research highlighting the detrimental effects of anxiety [13], we explicitly identify anxiety as an inhibiting psychological factor in university student’s ability to effectively engage in learning and contribute to the broader community.

Third, this study examines the mediating role of anxiety in the association between perceived environmental uncertainty and university students’ behavior, thereby elucidating the underlying psychological mechanism through which perceived environmental uncertainty influences university students’ academic engagement and prosocial behavior. The results show that perceived environmental uncertainty positively affects university students’ anxiety levels, which subsequently leads to decreased academic engagement and prosocial behavior. Thus, our contribution to the literature lies in uncovering anxiety as a crucial psychological mediator linking perceived environmental uncertainty to its downstream consequences.

Finally, we contribute to the uncertainty management research by highlighting the importance of intolerance of uncertainty in influencing anxiety stemming from perceived environmental uncertainty on academic engagement and prosocial behavior. Building on the existing literature on uncertainty management [11,16], we show that enhancing the ability to cope with uncertainty, such as reducing the intolerance of uncertainty, can help university students effectively navigate uncertain environments and mitigate the negative effects of anxiety. Our findings indicate that through anxiety reduction, the intolerance of uncertainty mitigates the negative influence of perceived environmental uncertainty on academic engagement. This suggests that students who are experiencing anxiety but have lower levels of intolerance of uncertainty are better equipped for learning activities. This finding emphasizes the significance of problem-focused coping strategies in mitigating high-anxiety situations induced by impending threats and uncertainties [41].

### 5.2. Practical Implications

Our findings also have significant practical implications for universities and their students. First, universities are urged to take steps to help students better cope with situations of environmental uncertainty and to mitigate the adverse effects on their mental well-being and subsequent behavior. It is evident that anxiety arising from perceived environmental uncertainty significantly affects both academic engagement and prosocial behavior. Therefore, our findings underscore the importance of anxiety in shaping university students’ behavior. Consequently, universities should actively support students in effectively managing anxiety by providing effective coping mechanisms and strategic training for navigating uncertain environments. In particular, providing training and lectures on topics such as resilience, stress management, and innovative learning approaches can be particularly beneficial.

Our study further shows that in the face of unprecedented threats, the use of uncertainty coping strategies, such as reducing an individual’s intolerance of uncertainty, can effectively mitigate the effects of anxiety. In particular, our research suggests that students with lower levels of uncertainty intolerance exhibit superior abilities to cope with ambiguous situations, thereby reducing the negative effects of anxiety and ultimately fostering greater engagement in academic pursuits. We advocate that universities offer training programs aimed at reducing students’ intolerance of uncertainty. For example, university faculty could facilitate this process by devoting more time to effective communication with students, thereby facilitating their accurate perception of environmental uncertainty and fostering the development of adaptive coping skills.

### 5.3. Limitations and Future Research Directions

This study confronts several limitations that necessitate consideration in future research. First, the cross-sectional design employed in this study precludes the establishment of robust causal relationships between the variables. Hence, the adoption of a longitudinal or experimental design is warranted to facilitate a rigorous analysis of the causal linkages between perceived environmental uncertainty, anxiety, academic engagement, and prosocial behavior among university students. Second, we did not collect data on participants’ experiences. Nonetheless, individual characteristics, such as their historical background and experiences, could potentially be crucial factors in predicting variations in perceived environmental uncertainty and associated mental health. Further research is required to delve into individual differences in the relationship between perceived environmental uncertainty, mental health, and behavioral patterns. Third, this study solely employs intolerance of uncertainty as a coping strategy to mitigate the indirect, negative impacts of perceived environmental uncertainty on corresponding behaviors through anxiety. Further research could potentially explore the intervention capabilities of other strategies to reduce both perceived environmental uncertainty and anxiety among university students.

### 5.4. Conclusions

In the face of increasing environmental uncertainty, it is imperative for researchers and managers to understand the impact of environmental uncertainty on the psychological well-being, decision making, and behavior of university students. This present study addressed this issue by elucidating the effects of perceived environmental uncertainty on anxiety, academic engagement, and prosocial behavior among university students. In particular, our findings show that perceived environmental uncertainty increases anxiety levels among university students, which subsequently decreases their involvement in academic engagement and prosocial behavior. Furthermore, we find that this negative effect, particularly the indirect relationship between perceived environmental uncertainty and academic engagement mediated by anxiety, can be mitigated if university students possess a strong ability to tolerate uncertainty.

## Figures and Tables

**Table 1:** Descriptive statistics and correlation matrix.

	Mean	S.D	1	2	3	4	5	6	7	8	9
1. Perceived environmental uncertainty	4.24	1.09	-								
2. Anxiety	4.12	1.54	0.17 *	-							
3. Academic engagement	3.86	1.08	0.06	−0.45 **	-						
4. Prosocial behavior	4.18	1.33	0.07	−0.20 **	0.41 **	-					
5. Intolerance of Uncertainty	4.49	0.97	0.09	0.43 **	−0.20 **	−0.04	-				
6. Gender	0.56	0.50	−0.09	0.06	−0.09	0.05	0.02	-			
7. Age	0.82	0.69	−0.04	0.04	−0.02	−0.02	0.03	−0.09	-		
8. Education level	0.64	0.54	−0.06	−0.04	0.14 *	0.07	0.05	0.04	0.76 **	-	
9. Major	0.31	0.46	0.08	−0.12	0.16 *	0.02	−0.14 *	−0.19 *	0.12	0.18 **	-

Note. *N* = 221. Gender (0 = male, 1 = female). Education level (0 = undergraduate, 1 = master, 2 = doctorate and above). Major (0 = humanities and social science, 1 = natural science). * *p* < 0.05. ** *p* < 0.01 (two-tailed).

**Table 2:** Confirmatory factor analysis.

Model	χ^2^	df	CFI	RMSEA	SRMR
Five-factor model: Proposed	1237.48	542	0.91	0.08	0.05
four-factor model: perceived environmental uncertainty and anxiety collapsed	1776.50	554	0.83	0.10	0.06
four-factor model: academic engagement and prosocial behavior collapsed	2408.07	554	0.74	0.12	0.09
One-factor model: All variables collapsed	3925.20	560	0.53	0.17	0.15

Note. CFI = comparative fit index; RMSEA = root mean square error of approximation; SRMR = standardized root mean squared residual.

**Table 3:** Results for path analyses.

Predictor	Dependent Variable											
	Model 1						Model 2					
	Anxiety		Academic Engagement		Prosocial Behavior		Anxiety		Academic Engagement		Prosocial Behavior	
	B	SE	B	SE	B	SE	B	SE	B	SE	B	SE
Gender	0.30	0.20	−0.18	0.13	0.14	0.18	0.29	0.19	−0.16	0.13	0.16	0.18
Age	0.30	0.22	−0.38 **	0.14	−0.23	0.20	0.35	0.20	−0.37 **	0.14	−0.22	0.20
Education level	−0.27	0.29	0.61 **	0.19	0.38	0.26	−0.43	0.27	0.58 **	0.19	0.35	0.26
Major	−0.38	0.22	0.17	0.14	−0.04	0.20	−0.18	0.20	0.20	0.14	0.01	0.20
Perceived Environmental Uncertainty	0.37 ***	0.07	−0.01	0.05	0.07	0.07	0.27 ***	0.07	−0.01	0.05	0.06	0.07
Anxiety			−0.29 ***	0.04	−0.21 **	0.07			−0.28 ***	0.04	−0.20 **	0.07
Intolerance of Uncertainty (IU)			−0.02	0.07	0.06	0.10	0.59 ***	0.10	−0.02	0.07	0.06	0.10
Anxiety × IU									−0.10 *	0.05	−0.10	0.07
R2	0.13		0.25		0.06		0.13		0.26		0.07	

Note. *N* = 221. B = unstandardized path coefficients; SE = standard error. * *p* < 0.05. ** *p* < 0.01, *** *p* < 0.001.

## Data Availability

The data presented in this study are available on request from the corresponding author.

## References

[1] Li J., Han X., Wang W., Sun G., Cheng Z. (2018). How social support influences university students’ academic achievement and emotional exhaustion: The mediating role of self-esteem. Learn. Individ. Differ..

[2] Arnett J.J. (2000). Emerging adulthood. A theory of development from the late teens through the twenties. Am. Psychol..

[3] Clayton S., Karazsia B.T. (2020). Development and validation of a measure of climate change anxiety. J. Environ. Psychol..

[4] Clayton S. (2020). Climate anxiety: Psychological responses to climate change. J. Anxiety Disord..

[5] Galway L.P., Field E. (2023). Climate emotions and anxiety among young people in Canada: A national survey and call to action. J. Clim. Chang. Health.

[6] Guerra O., Eboreime E. (2021). The Impact of Economic Recessions on Depression, Anxiety, and Trauma-Related Disorders and Illness Outcomes—A Scoping Review. Behav. Sci..

[7] Zheng S., Wu G., Zhao J., Chen W. (2022). Impact of the COVID-19 epidemic anxiety on college students’ employment confidence and employment situation perception in China. Front. Psychol..

[8] Caggiano G., Castelnuovo E., Kima R. (2020). The global effects of COVID-19-induced uncertainty. Econ. Lett..

[9] Yan D., Zhang H., Guo S., Zeng W. (2022). Influence of anxiety on university students’ academic involution behavior during COVID-19 pandemic: Mediating effect of cognitive closure needs. Front. Psychol..

[10] Brooks A.W., Schweitzer M.E. (2011). Can Nervous Nelly negotiate? How anxiety causes negotiators to make low first offers, exit early, and earn less profit. Organ. Behav. Hum. Decis. Process..

[11] Lind E.A., van den Bos K. (2002). When fairness works: Toward a general theory of uncertainty management. Res. Organ. Behav..

[12] Hirsh J.B., Mar R.A., Peterson J.B. (2012). Psychological entropy: A framework for understanding uncertainty-related anxiety. Psychol. Rev..

[13] Trougakos J.P., Chawla N., McCarthy J.M. (2020). Working in a pandemic: Exploring the impact of COVID-19 health anxiety on work, family, and health outcomes. J. Appl. Psychol..

[14] Hu J., He W., Zhou K. (2020). The mind, the heart, and the leader in times of crisis: How and when COVID-19-triggered mortality salience relates to state anxiety, job engagement, and prosocial behavior. J. Appl. Psychol..

[15] White H.A. (2022). Need for cognitive closure predicts stress and anxiety of college students during COVID-19 pandemic. Pers. Individ. Differ..

[16] Sharma P., Leung T., Kingshott R.P., Davcik N.S., Cardinali S. (2020). Managing uncertainty during a global pandemic: An international business perspective. J. Bus. Res..

[17] Carleton R.N. (2016). Into the unknown: A review and synthesis of contemporary models involving uncertainty. J. Anxiety Disord..

[18] Milliken F.J. (1987). Three Types of Perceived Uncertainty About the Environment: State, Effect, and Response Uncertainty. Acad. Manag. Rev..

[19] Sniazhko S. (2019). Uncertainty in decision-making: A review of the international business literature. Cogent Bus. Manag..

[20] Zayadin R., Zucchella A., Anand A., Jones P., Ameen N. (2023). Entrepreneurs’ Decisions in Perceived Environmental Uncertainty. Br. J. Manag..

[21] Lazarus R.S. (1991). Emotion and Adaptation.

[22] Lian H., Li J., Du C., Wu W., Xia Y., Lee C. (2022). Disaster or opportunity? How COVID-19-associated changes in environmental uncertainty and job insecurity relate to organizational identification and performance. J. Appl. Psychol..

[23] Fu S., Greco L.M., Lennard A.C., Dimotakis N. (2021). Anxiety responses to the unfolding COVID-19 crisis: Patterns of change in the experience of prolonged exposure to stressors. J. Appl. Psychol..

[24] Lazarus R.S., Folkman S. (1984). Stress, Appraisal, and Coping.

[25] Steimer T. (2002). The biology of fear- and anxiety-related behaviors. Dialog-Clin. Neurosci..

[26] Kouchaki M., Desai S.D. (2015). Anxious, threatened, and also unethical: How anxiety makes individuals feel threatened and commit unethical acts. J. Appl. Psychol..

[27] Kahn W.A. (1990). Psychological Conditions of Personal Engagement and Disengagement at Work. Acad. Manag. J..

[28] Schaufeli W.B., Salanova M., González-Romá V., Bakker A.B. (2002). The Measurement of Engagement and Burnout: A Two Sample Confirmatory Factor Analytic Approach. J. Happiness Stud..

[29] Eysenck M.W., Byrne A. (1992). Anxiety and susceptibility to distraction. Pers. Individ. Differ..

[30] Marks I.F., Nesse R.M. (1994). Fear and fitness: An evolutionary analysis of anxiety disorders. Ethol. Sociobiol..

[31] Rodell J.B. (2013). Finding Meaning through Volunteering: Why Do Employees Volunteer and What Does It Mean for Their Jobs?. Acad. Manag. J..

[32] Hirschberger G., Ein-Dor T., Almakias S. (2008). The Self-Protective Altruist: Terror Management and the Ambivalent Nature of Prosocial Behavior. Pers. Soc. Psychol. Bull..

[33] Dugas M.J., Gagnon F., Ladouceur R., Freeston M.H. (1998). Generalized anxiety disorder: A preliminary test of a conceptual model. Behav. Res. Ther..

[34] Brislin R.W., Lonner W.J., Berry J.W. (1986). The wording and translation of research instruments. Field Methods in Cross-Cultural Research.

[35] Waldman D.A., Ramirez G.G., House R.J., Puranam P. (2001). Does Leadership Matter? CEO Leadership Attributes and Profitability Under Conditions of Perceived Environmental Uncertainty. Acad. Manag. J..

[36] Watson D., Clark L.A., Tellegen A. (1988). Development and validation of brief measures of positive and negative affect: The PANAS scales. J. Personal. Soc. Psychol..

[37] Carleton R.N., Norton P.J., Asmundson G.J. (2007). Fearing the unknown: A short version of the Intolerance of Uncertainty Scale. J. Anxiety Disord..

[38] Byrne B.M. (2001). Structural Equation Modeling With AMOS, EQS, and LISREL: Comparative Approaches to Testing for the Factorial Validity of a Measuring Instrument. Int. J. Test..

[39] Muthén L.K., Muthén B.O. Mplus User’s Guide.

[40] Aiken L.S., West S.G. (1991). Multiple Regression: Testing and Interpreting Interactions.

[41] Fiske S.T., Morling B., Stevens L.E. (1996). Controlling Self and Others: A Theory of Anxiety, Mental Control, and Social Control. Pers. Soc. Psychol. Bull..

